# Complex Reaction Network Thermodynamic and Kinetic Autoconstruction Based on *Ab Initio* Statistical Mechanics: A Case Study of O_2_ Activation on Ag_4_ Clusters

**DOI:** 10.1021/acs.jpca.1c03454

**Published:** 2021-06-16

**Authors:** Weiqi Wang, Xiangyue Liu, Jesús Pérez-Ríos

**Affiliations:** Fritz-Haber-Institut der Max-Planck-Gesellschaft, Faradayweg 4-6, D-14195 Berlin, Germany

## Abstract

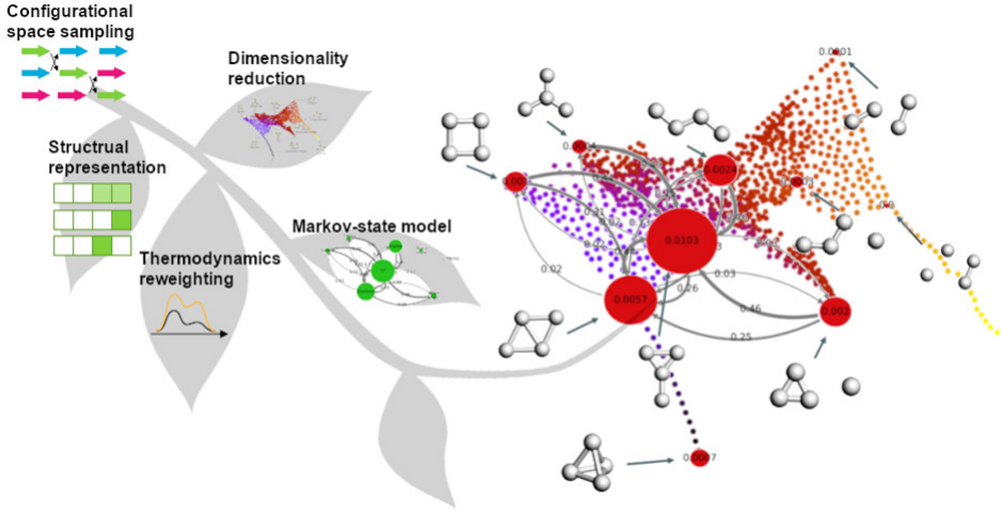

An approach based on *ab initio* statistical mechanics is demonstrated for autoconstructing complex reaction networks. *Ab initio* molecular dynamics combined with Markov state models are employed to study relevant transitions and corresponding thermodynamic and kinetic properties of a reaction. To explore the capability and flexibility of this approach, we present a study of oxygen activation on Ag_4_ as a model reaction. Specifically, with the same sampled trajectories, it is possible to study the structural effects and the reaction rate of the cited reaction. The results show that this approach is suitable for automatized construction of reaction networks, especially for non-well-studied reactions, which can benefit from this *ab initio* molecular dynamics based approach to construct comprehensive reaction networks with Markov state models without prior knowledge about the potential energy landscape.

## 1 Introduction

The chemical industry is transitioning to the so-called green chemistry, which relies on renewable reactants that produce minimal hazardous waste. Therefore, a sustainable industrial practice hinges on controlling and tailoring chemical reactions, which is the main focus of catalysis. Interestingly enough, even fundamental catalytic processes involve a complex reaction network depending on various reaction conditions. Therefore, a proper theoretical approach to the thermodynamics and kinetics of catalysis is required.^1−4^ The catalytic reaction network constructed based on microkinetic modeling can bridge atomic-scale properties with macroscopic observables of the reactions.^5−7^ Indeed, microkinetic models can be well established on top of elementary step properties, e.g., rate constants, obtained from first-principles calculations.^8,9^ Furthermore, the recent combination of machine-learning algorithms and first-principles calculations extends the range of applicability of the microkinetic modeling to more complex reaction networks.^10^

Traditionally, a theoretical framework treating catalytic reactions relies on identifying the reaction pathways by looking at the elementary steps toward the final product states. In particular, the reaction pathway is found through the minimum energy path (MEP) once transition states are identified. Then, the reaction rates can be calculated based on the transition state theory (TST)^11^ and microkinetic modeling, elucidating the reaction mechanisms.^12−15^ However, the success of this method depends on the ability to search for stationary geometries (e.g., intermediates and transition states) along the reaction pathway, which is a challenging and demanding task. Therefore, such an approach is hardly generalizable, and it becomes computationally prohibitive for complex systems. Furthermore, free energy calculations in a reaction network within the TST framework depend on the harmonic oscillator (HO) approximation,^16^ which is inaccurate at high temperatures because it neglects the translational and rotational motions of weak interactions between molecules and substrates.^17−19^ In the meantime, at high temperatures, the reaction pathway will most probably not follow the MEP, based on the transition state (active complex) hypothesis. On the other hand, there is a decent amount of work on using molecular dynamics methods in catalysis and surface reactions,^17,20−27^ providing more accurate descriptions of the free energy and reaction pathways at finite temperatures than HO-TST methods.

In this work, we demonstrate an approach from the active field of protein folding combined with *ab initio* molecular dynamics (AIMD) to construct reaction networks relevant to catalytic reactions. More specifically, AIMD with the replica exchange enhanced sampling method (REMD)^28^ is employed to sample the chemical space efficiently. Then, we build Markov state models (MSMs)^29,30^ by estimating the transition matrix between discrete states. The reaction network can therefore be automatically constructed from this approach in which all the relevant transitions between configurations can be included. Finally, we employ the transition path theory (TPT)^30,31^ method to calculate reaction rates between different configurations without requiring prior knowledge of the potential energy landscape.

Replica exchange enhanced sampling methods^28,32^ do not require preset reaction coordinates since the sampling is accelerated only by the temperature based on the equipartition theorem. In principle, REMD is not as efficient as explicit reaction coordinate methods (e.g., meta-dynamics,^33^ umbrella sampling^34,35^) when the reaction pathway is known for a specific reaction. However, a temperature-based enhanced sampling method, such as integrated tempering sampling^36,37^ and REMD, can comprehensively explore all the possible degrees of freedom for an unknown reaction. Therefore, its generalization to unknown reactions is superior to explicit reaction coordinate methods, which are not suitable to construct reaction networks automatically.

Usually, MD-based methods suffer from high computational cost.^7^ Therefore, in the present approach, we do not try to identify all the elementary steps in the reaction network but instead construct in one shot a comprehensive reaction network from the MD-sampled results. The memoryless properties of MSMs allow the study of long-term chemical kinetics in relatively short time trajectories. Therefore, massive parallelization of AIMD trajectories can be possible.

Generally, a reaction network consists of transitions going from reactants to products via intermediates. However, the definitions of reactants and products are not necessary for a reaction network since we focus on the transitions in the whole system between different configurations, which do not have to be stationary points on the potential energy surface. Based on the transition matrix, which can be considered as the transition probability between any pair of configurations in the phase space, MSMs can describe the stabilities and the transitions. Therefore, the reaction network from a specific reactant to a specific product is included naturally in the MSMs.

To obtain the reaction rate and reaction pathways for a specific reaction, the TPT is employed to analyze the MSMs. TPT is derived from transition path sampling (TPS)^38,39^ and is adequate for MSMs. In other words, explicit reaction coordinates and transition states are not required in the calculation of reaction rate by TPT. These features avoid the shortcomings in choosing the order parameters for the reaction coordinates and the approximation of rate constant calculation within the TST approach. TPT works as a statistical method on the transitions between Markov states to calculate any reaction network transition rate. Indeed, the reaction rate of a specific reaction and its corresponding weighted reaction pathways are readily obtained for a given reaction network.

To illustrate the current approach, we study a model catalytic reaction: oxygen activation on Ag_4_ clusters at finite temperatures. Silver is a commonly used catalyst for catalytic oxidation in many industrial chemical processes, for example, in ethylene epoxidation.^40^ In realistic reaction processes at finite temperatures, the formation of coexisting isomers, including transient metastable structures, may promote the activation of the ligand molecule on silver catalysts.^25,26,41^ In the meantime, the dynamically changing structure of a catalyst–ligand complex under operating conditions introduces additional complexity to the reaction network, which can be unforeseeable by human intuition. Using the *ab initio* REMD-based MSMs, we study the dynamically changing structures of Ag_4_ and their roles in promoting oxygen activation. In addition, using TPT analysis, we obtain the transitions which we are interested in to obtain the corresponding reaction rates and pathways. Finally, the dependency of the reaction rate on temperature is also studied.

## 2 Theory and Methodology

**Figure 1:**
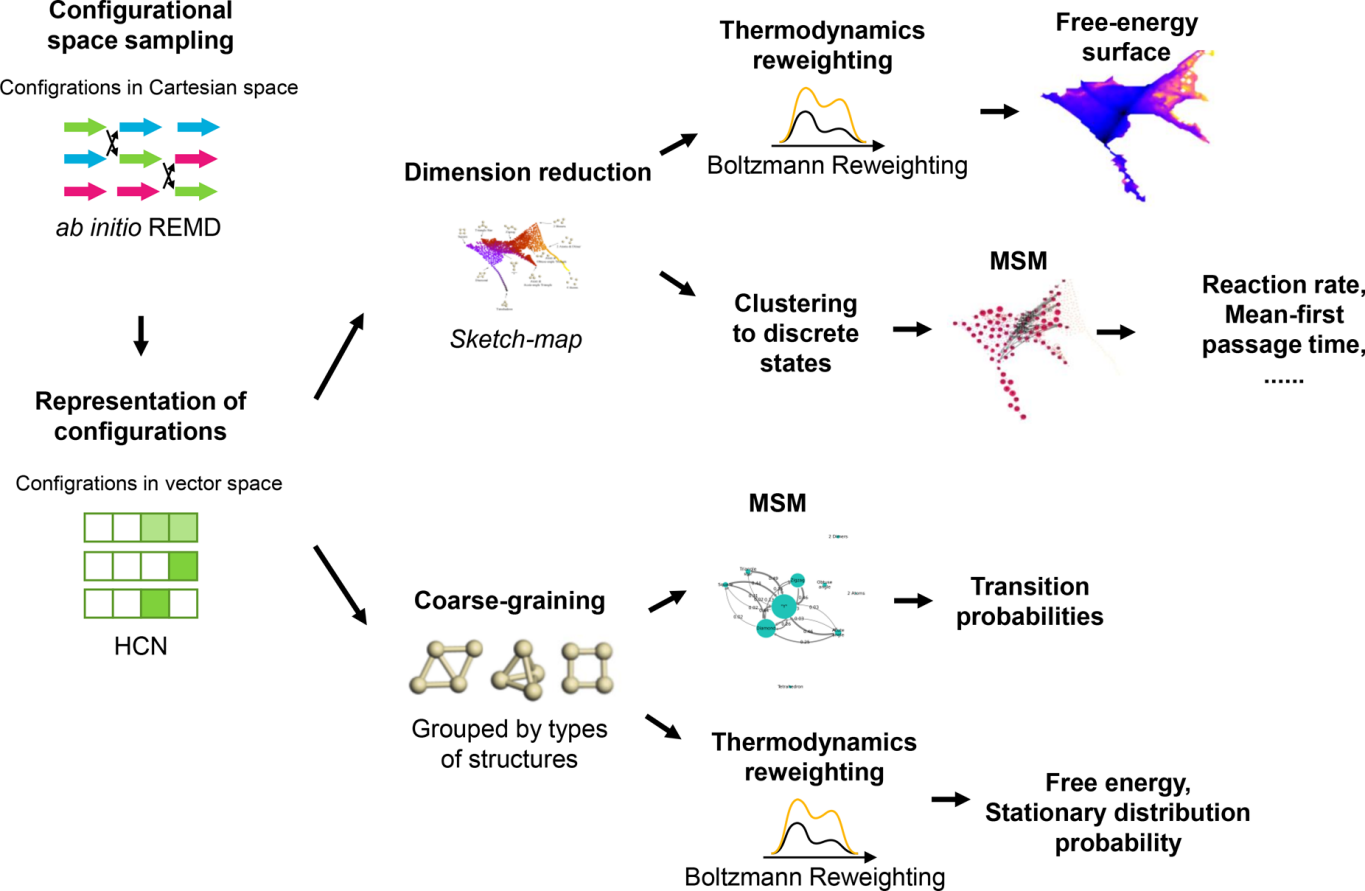
Scheme of the methodologies employed in this work. The configurational space is sampled by *ab initio* REMD and represented by HCN. Then the sampled configurations in HCN are either projected to two-dimensional space via sketch-map to construct detailed reaction networks about the transitions between different configurations or coarse-grained by typical structures to analyze the role of the Ag_4_ structure in promoting O_2_ activation.

The present approach relies on a proper sampling of the chemical space, which is done in this work by *ab initio* molecular dynamics. After an adequate representation of the chemical space is identified, different geometries are linked via the Markov state theory, and finally, the reaction rate is calculated within the transition path theory framework. Figure 1 shows the scheme of the methodology presented in this work. In this section, we present the three fundamental pillars of this theoretical approach.

### 2.1 Replica Exchange *Ab Initio* Molecular Dynamics (REMD)

*Ab initio* molecular dynamics is employed to sample the available chemical space efficiently. The motion of electrons and nuclei are separated under the Born–Oppenheimer approximation. The interactions between atoms are calculated on-the-fly by electronic structure methods (e.g., density functional theory). The dynamics of the atoms are given by Newton’s equation of motion. In particular, we work within the REMD framework^28,32^ in a canonical (NVT) ensemble. In this formalism, several trajectories at given temperatures are launched and exchanged during the time evolution following the Boltzmann probability satisfying the detailed balance condition, i.e.,
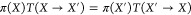
1Here, π(*X*) is the stationary distribution probability for the state *X*, and the transition probability *T*(*X* → *X*′) is calculated following the Metropolis criterion as
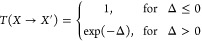
2where
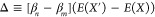
3 with *k*_B_ denoting the Boltzmann constant, and *T*_*i*_ stands for the temperature of the *i*th trajectory. *E*(*X*) represents the energy of the trajectory associated with the state *X*. It is worth noticing that high-temperature replica helps the fast convergence of low-temperature ones for rare events. Therefore, REMD is a robust, efficient, and appropriate method to calculate the dynamics of many-body systems involving chemical reactions.

### 2.2 Representation

The configurations sampled by REMD are generally represented by high-dimensional Cartesian coordinates. In general, it is necessary to find an invariant, unique, continuous, and general map from the Cartesian coordinates of the constituent atoms onto a Hilbert space.^42^ In recent years, various representations have been developed and successfully applied in chemistry and materials science.^42−46^ The choice of representations of molecules depends on the system at hand. For Ag_4_ clusters, the histogram of coordination number (HCN)^47^ is an appropriate method that can present different configurations in unique vectors.

For a system with *N* atoms, HCN gives the accumulated number of atoms, *h*_*i*_, for a specific coordination environment with coordination number CN as
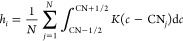
4where *c*_*j*_ is the coordination number of the *j*th atom obtained from the pairwise distances between atoms. *K*(*c*) is a function that specifies the fraction of the *j*th atom associated with the coordination number CN. For more details, we recommend the reader to look into eqs 6–9 of ref (47) and the references therein.

### 2.3 Free Energy Calculations

To calculate the free energy, the sampled configurations of a system need to be converted into thermodynamic states. Considering that the configurations are represented by high-dimensional vectors, it is convenient to employ a dimension reduction algorithm so that the resultant free energy surfaces can be easily understood. Sketch-map^48^ is a dimension reduction algorithm based on multidimensional metric scaling specially designed for dealing with molecular dynamics scenarios, e.g., the thermal fluctuation in the vicinity of the energetic basins or the poor sampling at the transition states.^47^ In sketch-map, a set of projections {*d*_*i*_} is generated from a set of high-dimensional landmark points {*D*_*i*_} by minimizing the stress function:
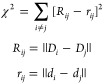
5where *R*_*ij*_ is a measure of the dissimilarity in the high-dimensional space, and *r*_*ij*_ is the Euclidean distance between their projections. *D*_*i*_ and *d*_*i*_ are the representations of configuration *i* in high-dimensional and low-dimensional space, respectively. The use of MD leads to short length scales due to the thermal fluctuations around local minima, which complicates the characterization of different configurations. To avoid this, sketch-map employs sigmoid filter functions *F*(*R*) and *f*(*r*) defined as
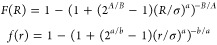
6that suppress short length scales. The parameter σ defines the length scales to divide local minima. *A* and *a* control the decay in the short-range, while *B* and *b* control the decay in the long-range. These parameters should be given before the projection. As a result, the stress function reads as
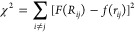
7

Finally, we use the multistate Bennett acceptance ratio (MBAR)^49^ approach to calculate free energies associated with each of the thermodynamic states. The basic idea of MBAR is to calculate the ratio between the partition functions of thermodynamic states obtained from different REMD replicas. Then, the dimensionless free energy reads as
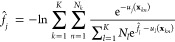
8which can be solved self-consistently. In this equation, *K* is the number of thermodynamic states, and *N*_*k*_ is the number of configurations in the thermodynamic state *k*. The Boltzmann factors are calculated by *u*_*j*_, which is the product of the inverse temperature and the potential energy of the configuration *i* with coordinate **x**_*kn*_. In practice, only the differences between free energies Δ*f̂*_*ij*_ = *f̂*_*j*_ – *f̂*_*i*_ are physically meaningful.

### 2.4 Reaction Rate Calculations

#### 2.4.1 Markov State Models

In a catalysis system, molecules or materials evolve continuously in its phase space, divided into reactant, transition state, intermediate, and product regions. The reactant region, intermediate region, and product region are the basins around the local minimum in which the system prefers to thermofluctuate in the potential energy (hyper)surface. However, the transition state region is less frequently visited by the system since transition states are saddle points in the potential energy (hyper)surface. Generally, transition states are studied to obtain information on the reaction rate from reactants to products. Therefore, the evolution of the system can be described by a set of trajectories in which the transitions from the reactants to the products can be extracted and analyzed.

MSMs are widely used in protein folding and in the study of macromolecules dynamics, both involving long time scales. Markov state models provide a framework to link the data of microscopic dynamics with macroscopic observations. In particular, Markov state models are inherently statistical methods;^29,30^ thus, relevant observables can be calculated as expectation values of the corresponding operators. Meanwhile, the memoryless nature of MSMs makes it possible to simulate many short-time trajectories instead of a long-time trajectory, so that the trajectories can be calculated in parallel. Furthermore, the evolution of the system over time can be retained in MSMs.

To map a catalysis system into Markov states, a proper clustering or discretization algorithm should be used. In this work, we use the regular-space clustering^50^ method, which forces the Markov states to be uniformly distributed in the reduced or phase space. Based on the Markov states obtained by the clustering method, the transition matrices can be calculated to describe the transitions between Markov states *i* and *j* in a thermodynamic equilibrium system via
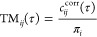
9Here π_*i*_ is the stationary probability of state *i*, *c*_*ij*_^corr^(τ) is the normalized time correlation function, and τ is the lag time.

#### 2.4.2 Transition Path Theory

TPT^30,31^ interprets the reactive paths statistically and calculates the relevant chemical observables (e.g., reaction rate) in terms of committor functions and transition probabilities. Based on the MSMs, reaction networks can be constructed on top of transition probabilities between Markov states. When the reaction networks are constructed, TPT calculates the flux of all possible transition pathways connecting the required reactant and the desired product through committors, which indicate the probabilities of transitions from a Markov state to the product or reactant. Based on the reaction networks, properties regarding the transition, such as the total flux, the mean first-passage time (MFPT), and reaction rate from the reactant to the product, can then be calculated.

## 3 Computational Details

In this work, electronic structure calculations of silver clusters with oxygen molecules are performed by an all-electron full-potential numerical atomic orbital basis set code, FHI-aims,^51^ with density functional theory (DFT) under the generalized gradient approximation (GGA) of the Perdew, Burke, and Ernzerhof (PBE)^52^ functional. “Tight” settings for the basis set and numerical integration grid are employed.^51^ Scalar-relativistic corrections have been applied,^51,53^ and vdW^*TS*^^54^ dispersion interactions are included by means of a *C*_6_[*n*]/*r*^6^ tail correction to the PBE energy, where the *C*_6_[*n*] coefficients are derived from the self-consistent electron density.

The AIMD simulations are performed in the NVT ensemble using a Bussi–Donadio–Parrinello thermostat^55^ with a time step of 0.002 ps. The REMD with 10 temperature replicas is performed to sample the configurations of silver clusters in the phase space. The temperatures of the replica are 200, 240, 280, 340, 405, 480, 570, 680, 815, and 970 K, while they exchange the temperatures every 10 MD steps. For each replica at the corresponding temperature, 125 000 configurations are sampled.

Ag_4_ clusters are represented by HCN which is sufficient to identify the configurations. Then, the sketch-map method is used to reduce the four-dimensional HCN vectors into a two-dimensional sketch-map space with 1000 samples chosen as so-called “landmarks”. The cutoff function defining the short, medium, and long pairs is given by setting the parameters as follows: σ = 0.25, *A* = 15, *B* = 1.5, *a* = 15, and *b* = 1.5. To calculate the free energies in two-dimensional sketch-map space, pymbar (a python implementation of MBAR) is employed to reweight the stationary distribution probabilities between temperature replicas of REMD. At last, the estimation of MSMs is performed by using PyEMMA packages^56^ with τ = 10 for this work.

## 4 Results

In this work, we address the most relevant physicochemical properties of oxygen activation on silver clusters by means of *ab inito* statistical mechanics based on the Markov state model presented above. In particular, our work focuses on answering two main questions:

- How does the structure of the silver cluster affect the oxygen activation?
- What are the oxygen activation rates along pathways at certain temperatures?

**Figure 2:**
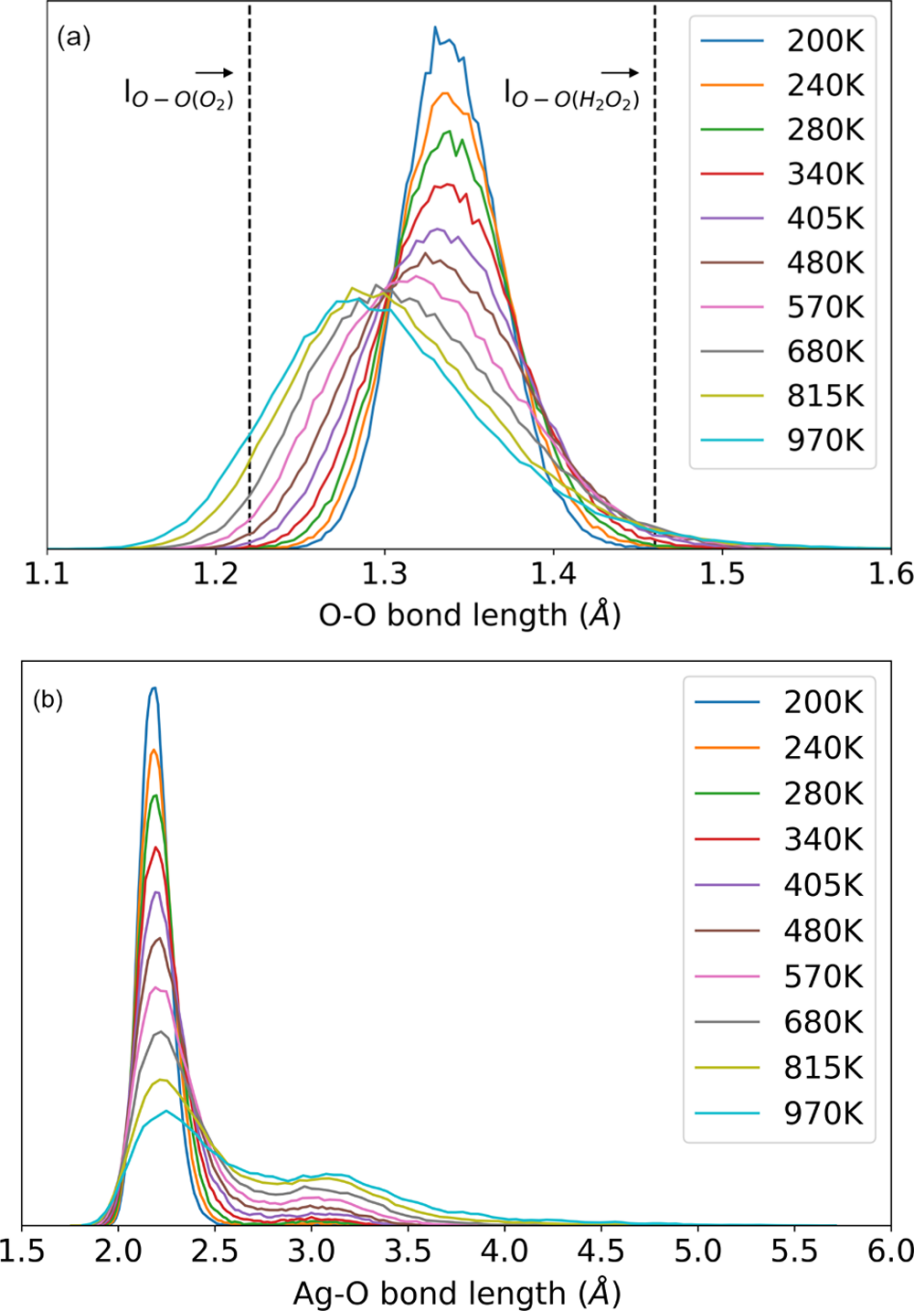
Histogram of bond length at different temperatures for O–O (a) and Ag–O (b) regarding oxygen adsorption on silver clusters. The PBE-optimized equilibrium O–O bond lengths in gas-phase O_2_ and H_2_O_2_ are 1.22 and 1.46 Å, respectively, shown in (a) as vertical dash lines.

The electronic ground state of O_2_ is a triplet, ^3^Σ_g_^–^, while the adsorbed O_2_ is in its singlet spin state ^1^Δ_g_. However, since we focus on the evolution of the Ag_4_O_2_ system after the chemisorption of O_2_ molecules, we assume that the O_2_ molecules are in the ^1^Δ_g_ electronic state in the simulations. As O_2_ adsorbs on silver, it experiences an elongation of the bond length due to a charge transfer between the molecule and the catalyst. In this process, the O–O bond is weakened from an O–O double bond to an O–O single bond in the Lewis picture of the molecular bond; i.e., the O_2_ is “activated” after adsorption on the silver clusters. To identify the activated species, it is necessary to define a criterion, and in this case, we use the O–O bond length. In particular, the configurations of Ag_4_O_2_ with O–O bond length longer than the one in H_2_O_2_ are identified as the activated states based on comparisons with peroxy compounds. The dissociated species are not discussed in this study since they are not observed, as shown in the distribution of O–O bond lengths as a function of temperature in panel (a) of Figure 2. Similarly, the distribution of Ag–O bond lengths is displayed in panel (b) of Figure 2, where we notice that 2.2 Å is the most probable Ag–O bond length in a wide range of temperatures. As a result, both O atoms of O_2_ prefer to bond with the Ag_4_ cluster, until one of the O atoms breaks the bond with the Ag_4_ cluster at higher temperatures.

After REMD simulations, the structures of Ag_4_ substrates in the Ag_4_O_2_ system are identified and represented by HCN vectors. To analyze the probability of activating O_2_ on Ag_4_ clusters with different structures, the sampled configurations are coarse-grained into 11 types of isomers based on the structure of the Ag_4_ substrates. The probabilities of activated O_2_ on these Ag_4_ isomers can then be calculated. With the aid of MBAR, the stationary distribution of different Ag_4_ substrate configurations is used to identify their actual contributions in activating O_2_ at a given temperature. To investigate the oxygen activation rates along different transitions, MSMs are built from the REMD results after dimension reduction by sketch-map. Based on these MSMs, TPT is employed to calculate the transition rates between a required reactant and a product visualized in sketch-map space.

### 4.1 Representation of Ag_4_O_2_ and Dimension Reduction

**Figure 3:**
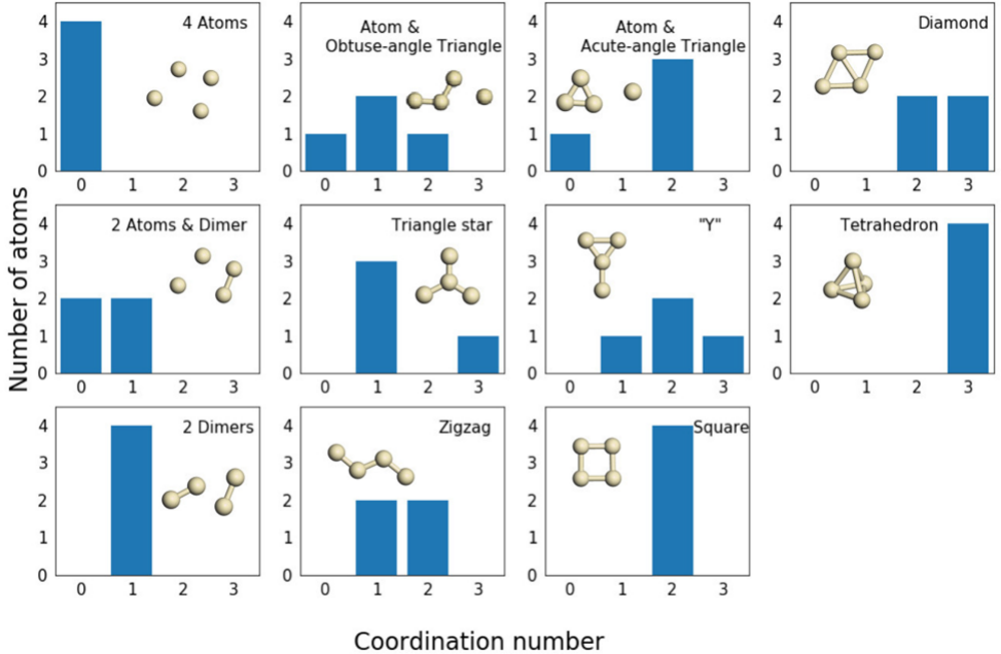
Eleven typical isomers of Ag_4_ and their HCN vectors.

Ag_4_ clusters are usually represented with 12-dimensional Cartesian coordinates. However, as introduced above, Cartesian coordinates are not invariant upon rotation and translation, and they are not size intensive. In this work, we use four-dimensional HCN to represent the structures of Ag_4_ substrates in the Ag_4_O_2_ system. As shown in Figure 3, 11 typical isormers of Ag_4_ are represented in HCN vectors, according to which the MD-sampled configurations can be coarse-grained to 11 groups based on the structural similarities.

**Figure 4:**
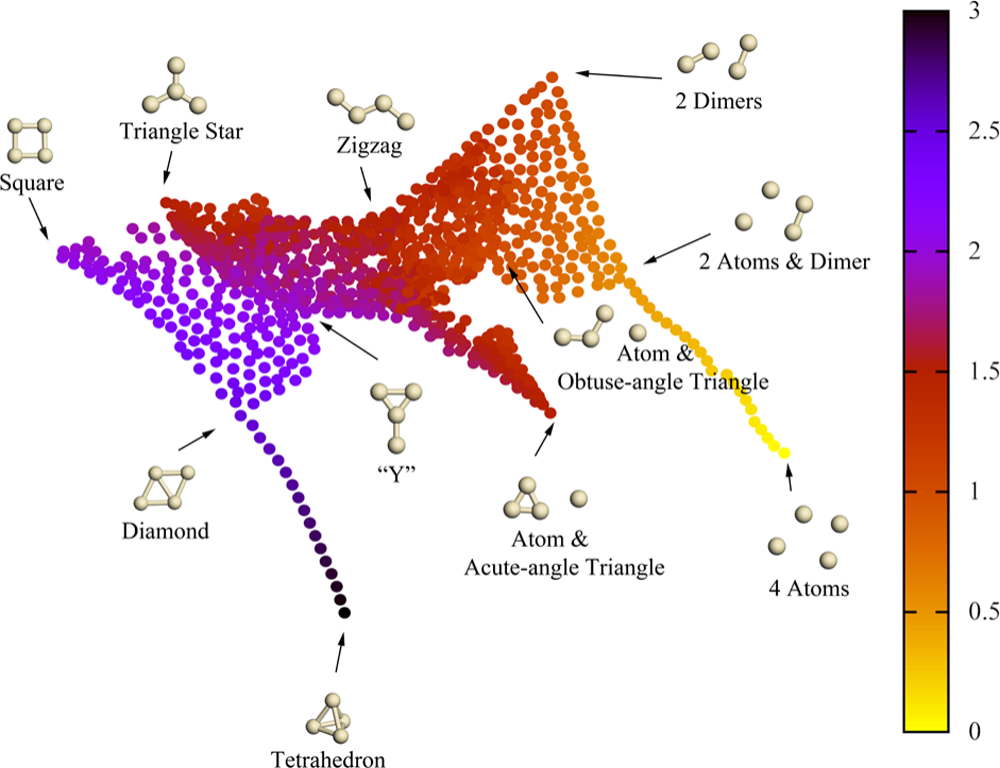
One thousand landmarks of Ag_4_ substrates mapped into a two-dimensional sketch-map space. Each dot in the plot indicates a configuration, while the color shows the average coordination number per atom of the configuration. In this reduced two-dimensional space, abscissas and ordinates of points are nonlinearly projected from a given high-dimensional space. The distances between configurations reveal their similarity. The arrows indicate the positions of relevant Ag_4_ structures in the two-dimensional space.

For the analysis of the oxygen activation rates along different transitions, the dimensionality reduction algorithm sketch-map is fed with the HCN vectors so that the Euclidean distances between different configurations in HCN are calculated. One thousand samples of Ag_4_ substrates are chosen as so-called “landmarks” by the minmax algorithm to ensure that they distribute in phase space uniformly. These landmarks are nonlinearly projected into a two-dimensional space afterward. The two-dimensional sketch-map space and the landmarks are shown in Figure 4, where 11 typical isomers of Ag_4_ substrates are labeled with their structures. By looking at the conversion of the colors, which express the average coordination number of the Ag atom in Ag_4_ substrates, we notice that the nonlinear projection from four-dimensional space to two-dimensional space is smooth and reasonable.

### 4.2 The Role of the Configurations of the Ag_4_ Substrate in Oxygen Activation

In this section, we try to address how the structures of the Ag_4_ substrate affect the activation of molecular oxygen. On one hand, O_2_ activation is related to the charge transfer from the substrate to the antibonding orbitals of O_2_ to weaken or even break the O–O bond. On the other hand, the Ag_4_ substrate shows various chemical activities with different structures due to the local environments of Ag atoms. Thus, the probability of O_2_ activation on the Ag_4_ cluster depends on the temperature.

#### 4.2.1 The Stability of Ag_4_O_2_ Configurations with Different Ag_4_ Substrates

**Figure 5:**
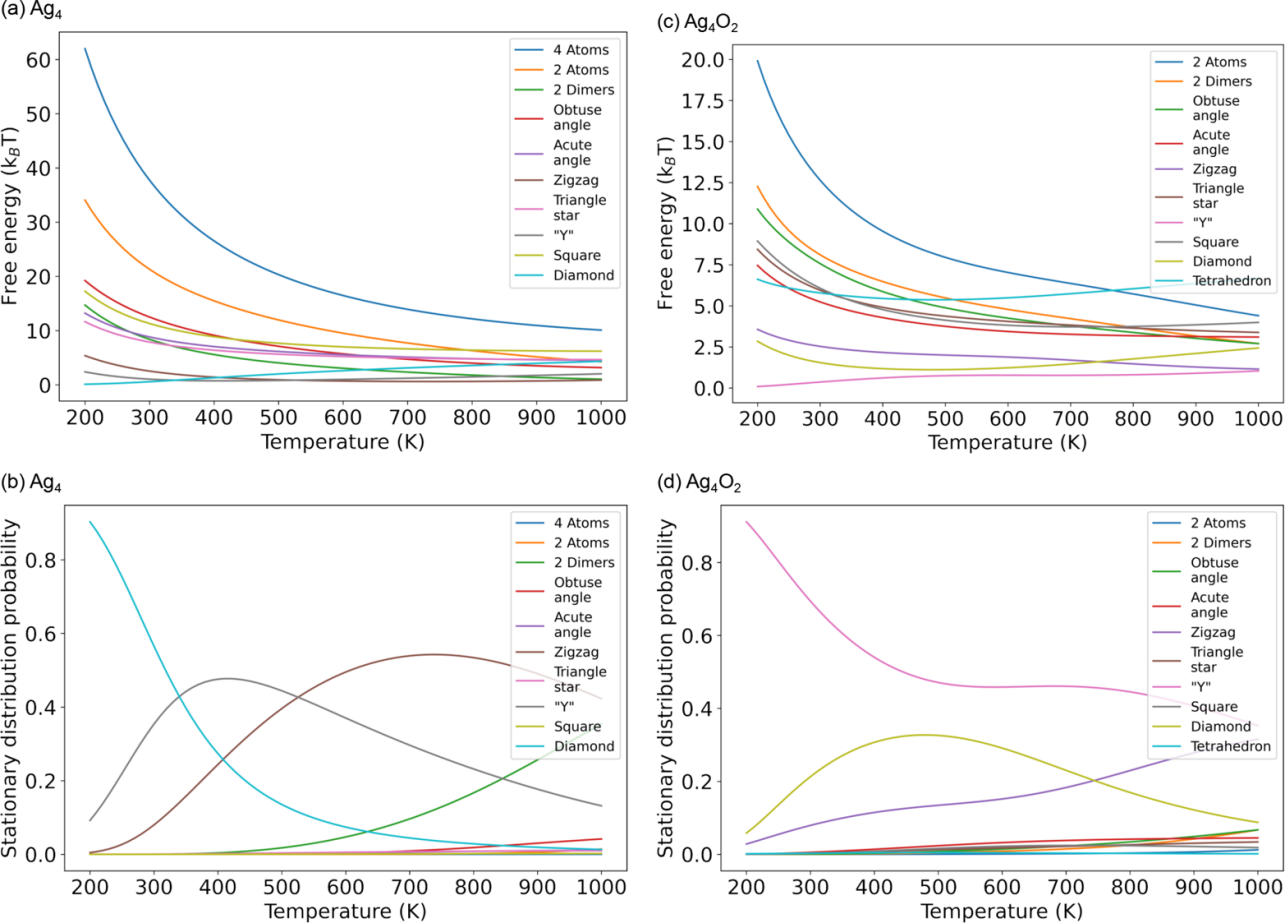
(a) Temperature dependency of the configurational free energies of Ag_4_ coarse-grained isomers without O_2_ in the gas phase. (b) Temperature dependency of the stationary distribution probability of Ag_4_ coarse-grained isomers without O_2_ in the gas phase. (c) Trends of the free energies of coarse-grained Ag_4_O_2_ isomers characterized by the Ag_4_ substrates changing with the temperature. (d) Trends of the stationary distributions probability of coarse-grained Ag_4_O_2_ isomers changing with the temperature.

At finite temperatures, various structural configurations of Ag_4_O_2_ will form an equilibrium ensemble, consisting of both stable and metastable states having different abilities to activate molecular oxygen. Using the representation exposed in the previous section based on 11 isomers, one can identify the temperature-dependent stability of Ag_4_O_2_ isomers with the corresponding Ag_4_ substrates, depending on the Ag_4_ characteristic structures. Indeed, the free energies and the stationary distribution probabilities of these 11 coarse-grained isomers show different temperature dependencies, as shown in Figure 5. More specifically, the relative stabilities of isomers strongly depend on the temperature. Panels (a) and (b) of Figure 5 show the free energies and stationary distribution probabilities of Ag_4_ clusters in the gas phase, while panels (c) and (d) are the results after the O_2_ adsorption. Indeed, we observe significant differences of Ag_4_ clusters’ relative stabilities before and after O_2_ adsorption. For example, the “4 atoms” structure is absent in the Ag_4_O_2_ system but observed in the Ag_4_ system. Without the adsorption of O_2_, 4 atoms is always the most unstable state at all temperatures. At low temperatures, the diamond state is the stablest isomer, while the Y state is the stablest isomer when the temperature becomes higher, and the zigzag state is the stablest isomer at the highest temperatures studied. We also notice that the stability of “2 dimers” quickly becomes higher with increasing temperature. These differences suggest that the O_2_ molecule can stabilize the Ag_4_ cluster in a configuration with a medium coordination number at the same time that the molecule is activated. However, after O_2_ is adsorbed on Ag_4_ clusters, the relative stabilities of A_4_ clusters become very different. From low to high temperatures, the Y structure is always the stablest state. As the temperature increases, the Y, diamond, and tetrahedron structures become less stable, and other structures become more stable. The dissociated state “2 atoms” is the most unstable state at low temperatures, while tetrahedron is the most unstable state at high temperatures.

#### 4.2.2 The Probability of O_2_ Activation on Different Ag_4_ Substrates

The distribution of configurations of silver clusters depends on the temperature, and the catalytic activities depend on the configurations. Consequently, the probability of O_2_ to be activated depends on the temperature. Therefore, it is important to study the activation probability of O_2_ at finite temperatures. Here, we take the Ag_4_O_2_ system at 570 K as an example to illustrate how the distribution of different configurations plays a role in oxygen activation and how to analyze their contribution by the present approach.

**Figure 6:**
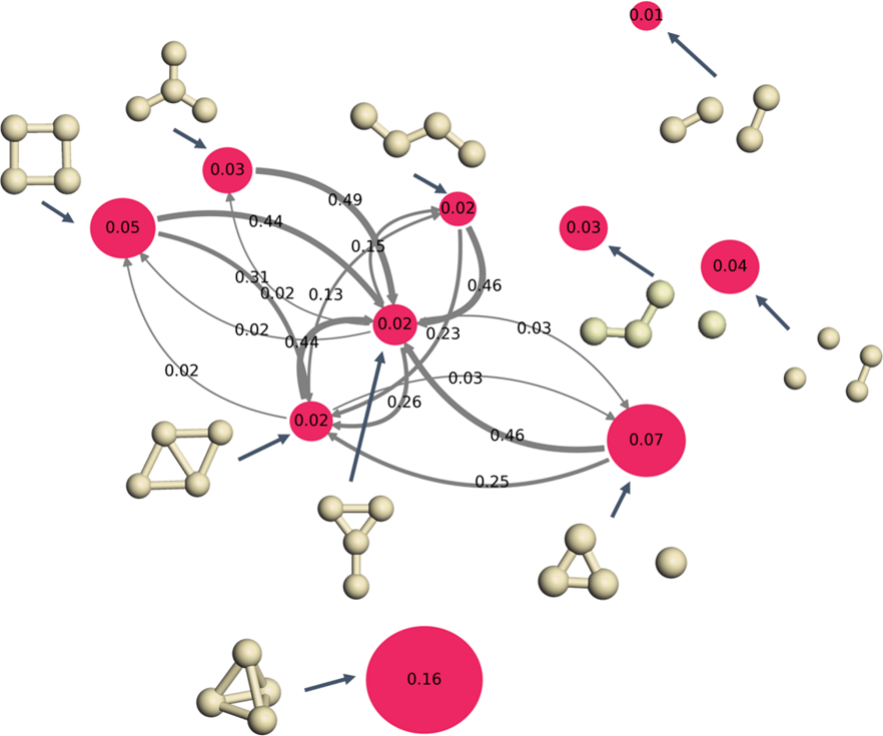
Transition probabilities between configurations shown as the arrows in the plot for the Ag_4_O_2_ system. The sizes of the nodes indicate the probability of O_2_ activation on relevant Ag_4_ structures.

Adsorption of O_2_ on Ag_4_ clusters is mediated by electron transfer from Ag_4_ to the antibonding π orbitals of O_2_. It is expected that some isomers of the silver cluster are more prominent to the electron transfer reaction than others, and, as a consequence, the O_2_ activation probability on Ag_4_ clusters with different configurations can be very different. To illustrate such dependency we display in Figure 6 the probability of O_2_ activation on the most relevant Ag_4_ isomers. As a result, tetrahedron, acute angle, and square isomers show the highest inherent O_2_ activation activity, whereas 2 dimers, Y, and zigzag activities are the lowest. In addition, we notice that the high-activity configurations are not necessarily the configurations with low Ag coordination numbers. For example, the coordination number of Ag in tetrahedron is higher than in the low-activity structures diamond and Y. This suggests that the activity of the Ag_4_ clusters can not be surmised by the coordination numbers of Ag atoms. For oxygen activation, the Ag_4_ configurations which can promote more electron transfer to the oxygen molecule will be more active. Ag atoms with more dangling bonds are usually more electronegative and can resist the processes of electron transfer. Additionally, the Ag_4_ clusters may stretch the O–O bond at finite temperatures due to their dynamically changing structures. Therefore, an unstable configuration with a larger range of structure distortion can also promote oxygen activation.

**Figure 7:**
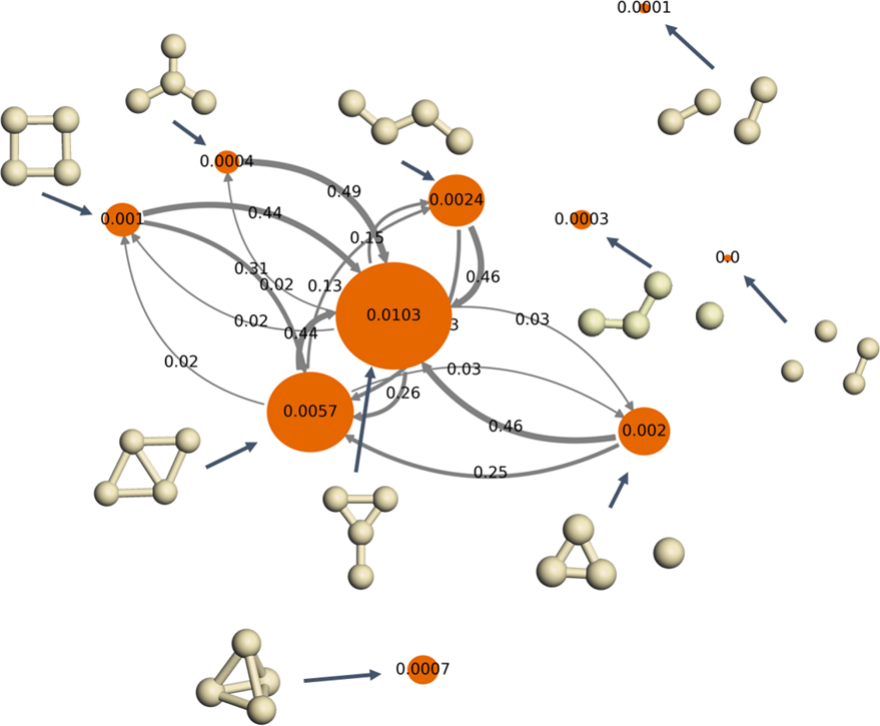
Normalized activation probability on the isomers of Ag_4_. The orange nodes label the isomers, and the numbers on them indicate the probability of O_2_ activation on Ag_4_. The arrows connect these isomers during the reaction with a transition probability given by the arrows’ numbers.

At first glance, tetrahedron, acute angle, and square contribute the most in activating O_2_. However, when the stability of the Ag_4_ isomers is taken into account, a new scenario emerges, as shown in Figure 7, where the normalized O_2_ activation probability on the isomers is shown. The numbers shown on the colored nodes correspond to the normalized activation probability of O_2_ molecules on each isomer, calculated by the ratio of the number of activated O_2_ molecules with respect to the total number of O_2_ molecules sampled at 570 K. At this temperature, owing to the high stationary distribution probabilities of zigzag, Y, and diamond configurations, these configurations contribute the most to O_2_ activation, despite the fact that the inherent O_2_ activation probabilities on these three isomers are relatively low. In particular, the normalized O_2_ activation probabilities on diamond, Y, and zigzag isomers are 0.57%, 1.03%, and 0.24%, respectively. On the contrary, even though tetradedron, acute angle, and square have very high inherent O_2_ activation activity, the normalized O_2_ activation probabilities on these isomers are only 0.07%, 0.2%, and 0.1%, respectively.

In realistic processes, the reactants, such as ethylene molecules, may react only with O_2_ activated on Ag_4_ isomers with particular configurations. In the meantime, the system is in a nonequilibrium state where Ag_4_ isomers with particular configurations are constantly consumed. As a result, the transition probabilities from other configurations to the consumed configurations promote the reaction. In Figure 7, the transition probabilities from one configuration to another are shown as the numbers above the arrows, while the widths of the arrows are proportional to the transition probabilities. At 570 K, we observe significant transitions between diamond, Y, zigzag, and square, triangle star, and atom and acute angle. As discussed above, square, triangle star, and atom and acute angle have the highest O_2_ inherent activation activity, but their normalized activation probability is lower than that of diamond, Y, and zigzag, which are the stablest isomers at 570 K. The high transition probabilities between these configurations can promote the reaction toward the product, leading to higher apparent activities of the consumed configurations.

### 4.3 The Kinetics of O_2_ Activation on Ag_4_

Considering the complexity of a reaction with dynamically changing structures, the MFPTs, weights, and temperature-dependency of reaction pathways are crucial to the understanding of a reaction. In this section, we first take 570 K as an example and study the free energy surface and the reaction network of O_2_ activation on Ag_4_. Then, we investigate the behavior of a given transition with temperature to elucidate the impact of the temperature of O_2_ activation on Ag_4_.

#### 4.3.1 The Reaction Networks at 570 K

**Figure 8:**
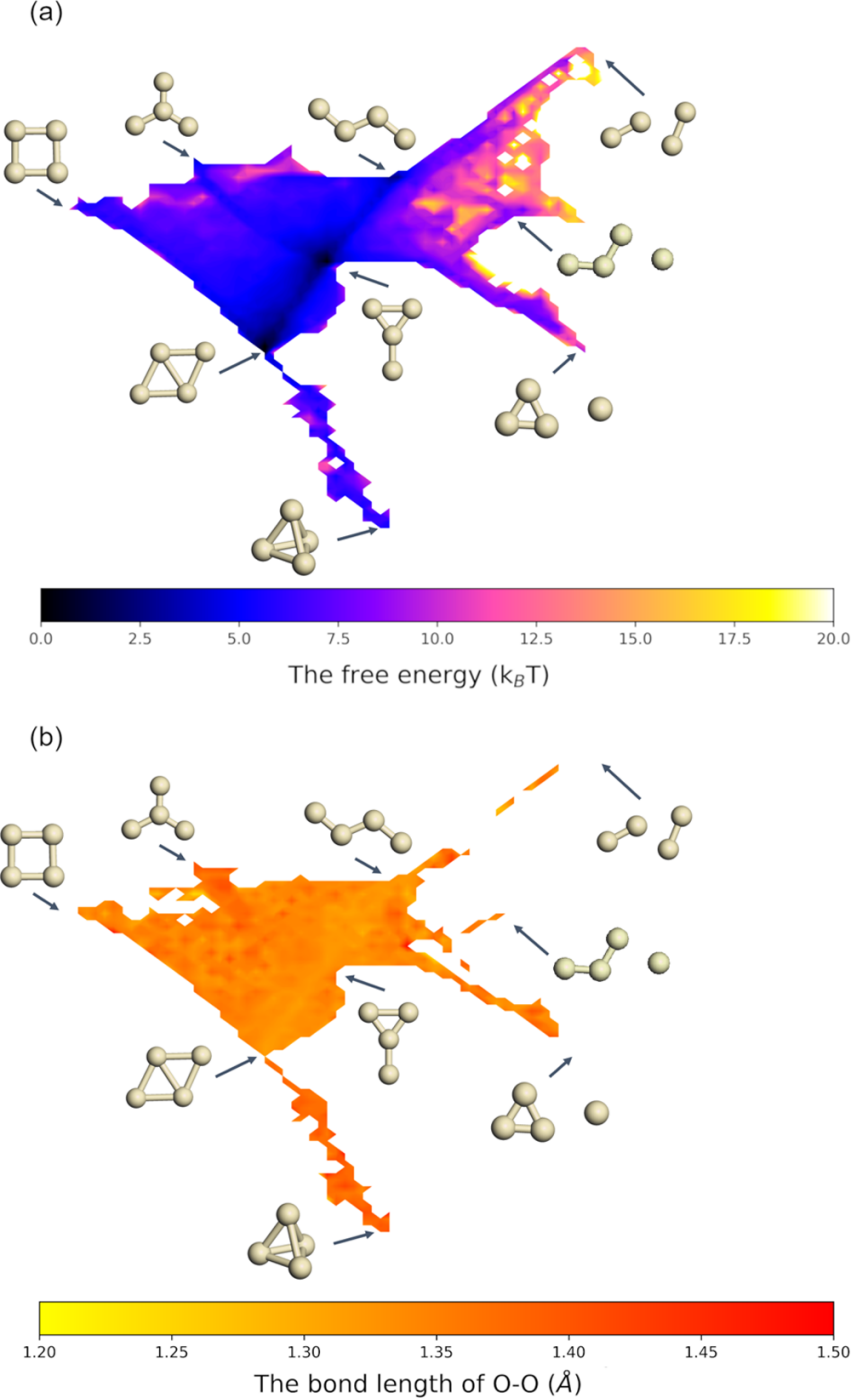
(a) Free energy surface of Ag_4_O_2_ at 570 K in the two-dimensional sketch-map space based on the structures of their Ag_4_ substrates. (b) The heat map of O–O bond lengths in the same two-dimensional space.

At finite temperatures, transitions between different stable and metastable structures can be massive, and the characterization of these transitions requires detailed knowledge about the free energy surface beyond the coarse-grained free energy study shown above. To this end, before exploring the free energy surface, we employ sketch-map to map the high-dimensional representations into a two-dimensional space based on the HCN of different Ag_4_ configurations. The resultant two-dimensional free energy surface of the Ag_4_–O_2_ system is shown in panel (a) of Figure 8, where it is noticed that diamond and Y isomers have the lowest free energy, which agrees with the coarse-grained results discussed in the previous section. There is a relatively stable area with low free energies between diamond and Y, suggesting massive transitions between these two configurations. However, looking at the heatmap of O–O bond length (projected into the same two-dimensional sketch map as the free energy) displayed in panel (b) of Figure 8, the O_2_ activation ability of this area is low. As discussed before, the transitions between configurations can be important to promote the activation of O_2_. Indeed, to activate O_2_, it is crucial to visit high-activity configurations such as triangle star and tetrahedron.

**Figure 9:**
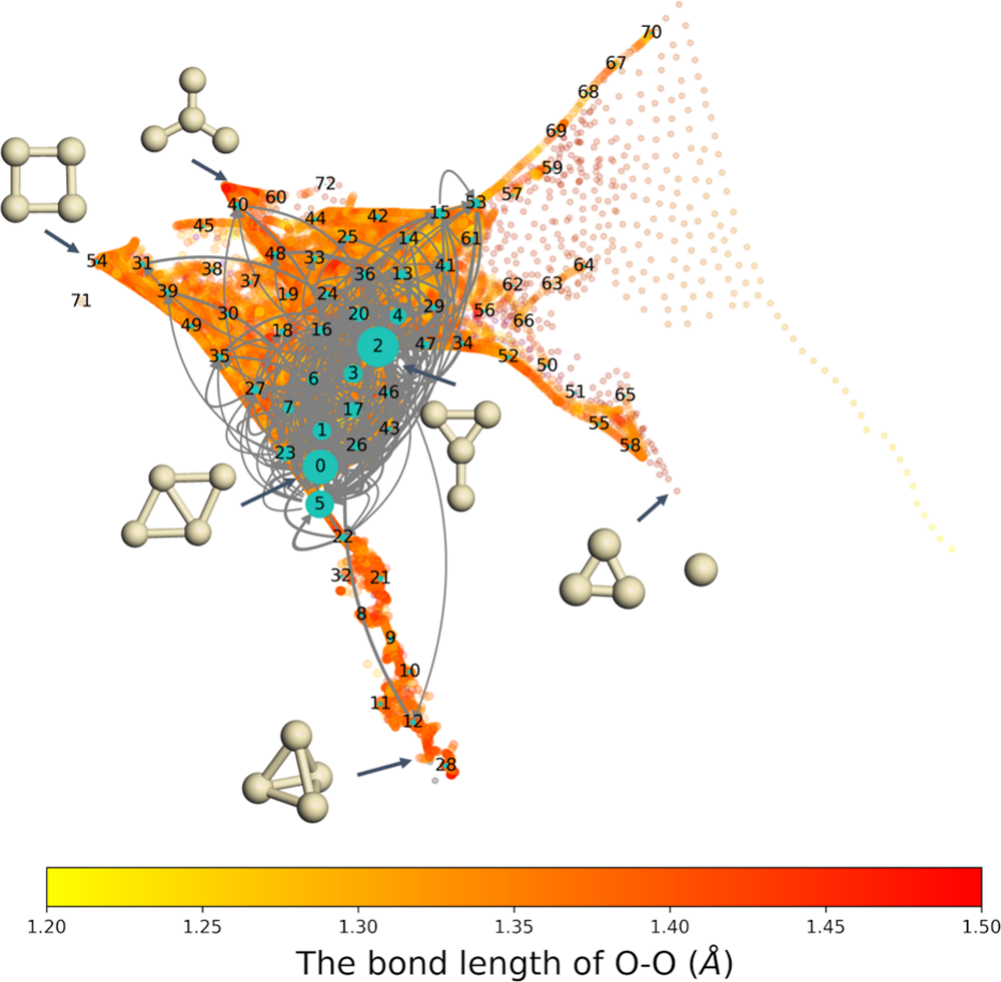
Transitions between different Markov states. The states are labeled with numbers, the size of cyan nodes shows the corresponding stationary distribution probabilities, and the heat map shows the O–O bond length. Both the transition network and the heat map are mapped into the sketch-map space of the Ag_4_ substrate. Although, in principle, transitions exist between all Markov states, only the ones with transition probability larger than 0.5% are shown.

To identify these transitions, we construct the MSMs shown in Figure 9 by uniformly partitioning the configurations up to 100 Markov states in the free energy surface. Since the sketch-map algorithm reduces the dimension of configurations based on structural similarity, configurations with similar structures are clustered into the same Markov states. Our results show that transitions between Markov states located around diamond and Y contribute the most to the transitions at this temperature. Although O_2_ can not be efficiently activated in the area around diamond and Y, we observe transitions between the low free energy states (around diamond and Y states) and the high free energy states (around acute angle, triangle star, and tetrahedron) with high activities.

**Figure 10:**
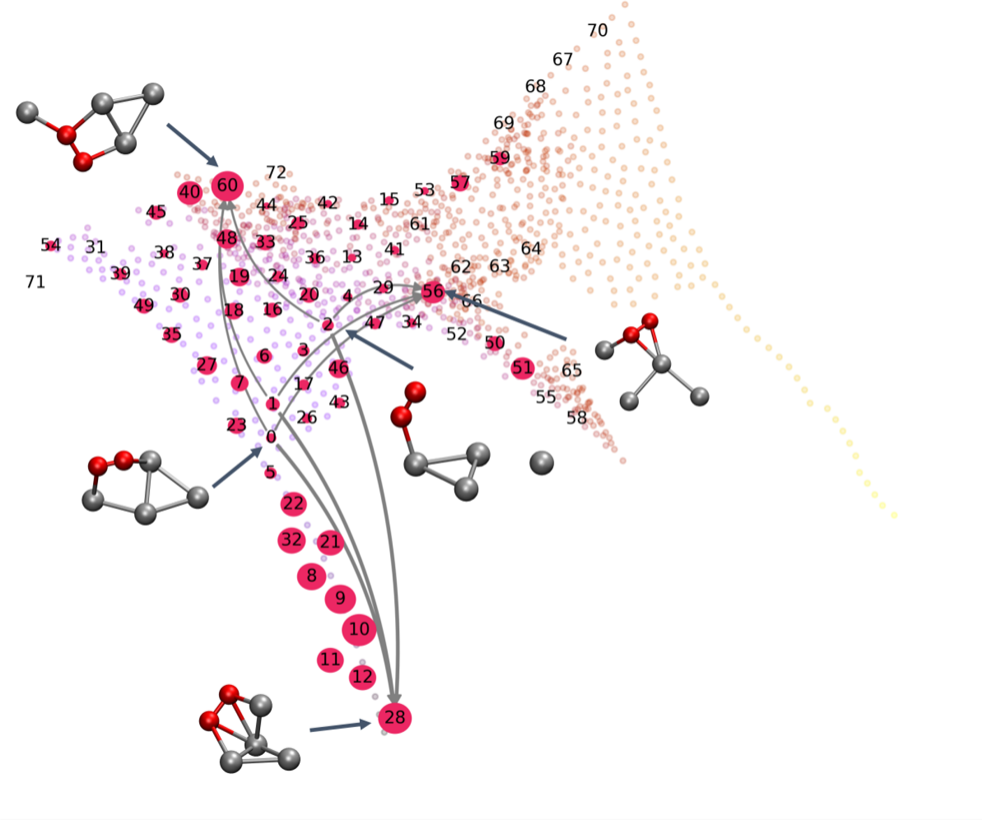
Transitions between three selected initial states (labeled as 0, 1, and 2) with low O_2_ activation probabilities and three final states (labeled as 28, 56, and 60) with high O_2_ activation probabilities. The size of the magenta nodes stands for the O_2_ activation probability. The corresponding MFPT of the transitions are summarized in Table 1. The transition pathways between state 0 and state 28 are summarized in Table 2.

To further investigate the transitions, we identify three low-activity states as the initial states and three high-activity states as the final states. The reaction rates of these nine transitions are calculated by TPT. The three initial states are labeled as 0, 1, and 2 in Figures 9 and 10, with Ag_4_ substrates having structures between diamond and Y. The three final states are labeled as 28, 56, and 60 in Figures 9 and 10. The structures of Ag_4_ substrates of state 28 and 56 are close to tetrahedron and triangle star, respectively, while the Ag_4_ substrate in state 60 is an intermediate structure around Y, zigzag, and atom and acute-angle triangle, on which the probability of activating the O–O bond is relatively high. The MFPT given by the inverse reaction rate of a transition from an initial state to a final state is summarized in Table 1. In this table, it is noticed that the reaction rates of these transitions depend mainly on the final states, and the reactions to state 28 are the fastest ones. Indeed, we have studied different transition pathways from state 0 to state 28 by calculating the reaction flux, as summarized in Table 2. The pathways “0 → 2 → 28” (23% flux) and “0 → 28” (16% flux) dominate the transition from state 0 to state 28. Therefore, for the transition from state 0 to state 28, the rate of O_2_ activation is mainly dominated by transitions with fewer intermediate states in their transition pathways.

**Table 1:** Summary of the Transitions between Three Selected Initial States (Reactant, Labeled as 0, 1, and 2 in Figure 9 and Figure 10) and Three Final States (Products, Labeled as 28, 56, and 60 in Figure 9 and Figure 10)

reactant	product	MFPT (ps)
0	28	11.14
0	56	36.23
0	60	42.76
1	28	11.15
1	56	36.24
1	60	42.76
2	28	11.14
2	56	36.23
2	60	42.78

**Table 2:** Transition Pathways with Corresponding Weights in the Total Reaction Flux from State 0 to State 28 Shown in Figure 10

transition pathways	weight
0 → 2 → 28	23%
0 → 28	16%
0 → 23 → 28	5%
0 → 5 → 28	4%
0 → 1 → 28	2%
0 → 5 → 2 → 12 → 28	2%
0 → 3 → 28	2%
0 → 17 → 28	2%
0 → 15 → 28	2%

#### 4.3.2 The Temperature Dependency of O_2_ Activation on Ag_4_

**Figure 11:**
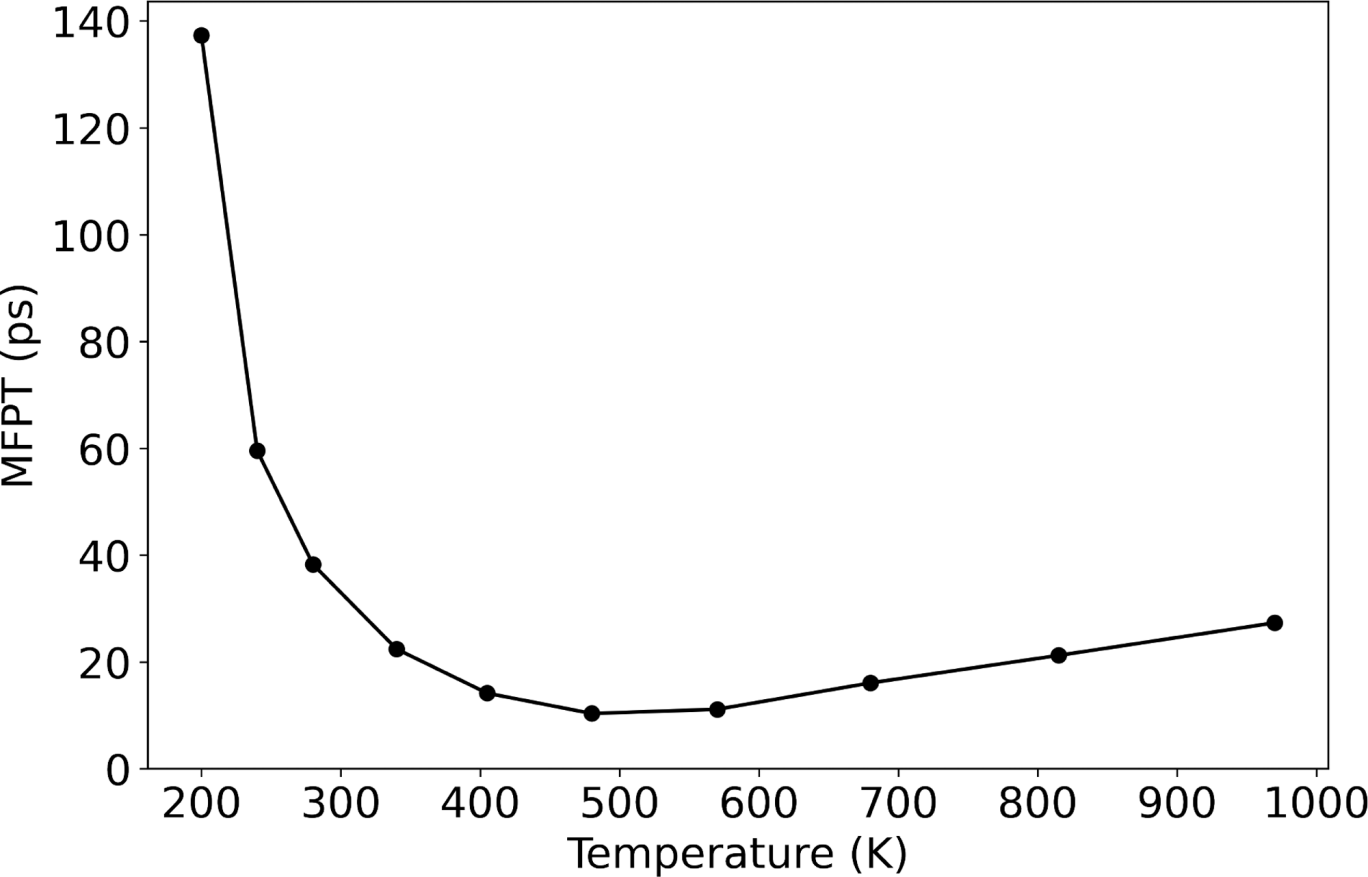
MFPT from O_2_ nonactivated state 0 to activated state 28 as a function of temperature.

In general, the reaction rate depends on the temperature, and for the case of a complex reaction, such dependency may be complicated. An example of this is displayed in Figure 11, where we show the MFPT of O_2_ activation on Ag_4_ from state 0 to state 28 as a function of temperature. This figure shows that the reaction rate reaches a maximum at a temperature ∼500 K and diminishes for higher or lower temperatures. However, the reaction rate toward lower temperatures shows a steeper trend than the one for higher ones. This behavior is related to the fact that the system is affected by both its thermodynamic and dynamic properties. At relatively low temperatures, a small rise in temperature dynamically increases the probability of overcoming the reaction barriers, leading to a higher apparent reaction rate. However, thermodynamically the probability of a high-activity species, which is the tetrahedron state in this case, becomes lower at very high temperatures, leading to a reduction of the apparent reaction rate.

## 5 Conclusions

In this work, we demonstrate an approach based on *ab initio* statistical mechanics to autoconstruct complex reaction networks in three steps: (1) REMD is used to sample the phase space efficiently. (2) A representation of the system should be flexible, depending on the study of the reaction at hand. (3) MSMs are constructed from which the reaction networks can be analyzed. Since the *ab initio* molecular dynamics can automatically search the phase space, prior knowledge about the energy landscape is not required. Almost all relevant transitions of the reaction can be preferentially sampled because AIMD simulates the reaction at operational conditions. Meanwhile, the thermodynamic and kinetic properties of the transitions between relevant configurations are also obtained.

To illustrate the capabilities and flexibility of this approach, we present a case study of a model system: O_2_ activation on Ag_4_ clusters. We discuss this system from two aspects, including the effect of the Ag_4_ structures on O_2_ activation and the kinetics of O_2_ activation. As a result, we find that the activation probability of O_2_ is determined not only by the inherent activity of Ag_4_ substrates but also by the thermodynamic stability of configurations, based on the stationary distribution probability of coarse-grained states at certain temperatures. The MFPTs of nine chosen transitions between selected reactants and products are calculated to investigate the reaction rate of the O_2_ activation. For one of these reaction pathways, we discuss in detail about the dominant transitions that contribute to the reaction flux and the temperature dependency of the reaction rate.

Compared with the HO-TST-based microkinetic modeling, in this approach, the screening of kinetic processes and the calculations of corresponding thermodynamic properties are simulated by Newtonian dynamics in phase space through first-principle calculations. The complex reaction network is constructed by MSMs automatically instead of microkinetic modeling. The advantages of using AIMD-based MSMs include the following: (1) AIMD can naturally take lateral interactions, anharmonic effects, and dynamic relaxation of structures into account, and (2) MSMs do not require rate-determining steps approximation and steady-state approximation. Although AIMD is the cost bottleneck, employing the enhanced sampling, reweighting method, or machine-learning potentials^57,58^ can reduce the workload of the simulation and extend the simulation to multiple operational conditions. Therefore, this approach is suitable for constructing complex reaction networks relevant to reactions automatically.

## Supporting Information Available

Method details, including the calculation of the histograms of coordination numbers, dimension reduction, Markov state models, and transition path theory (PDF)
