# Supplementary material for: Complex Reaction Network Thermodynamic and Kinetic Autoconstruction Based on Ab Initio Statistical Mechanics: A Case Study of O2 Activation on Ag4 Clusters
Source: J Phys Chem A. 2021 Jun 16;125(25):5670–80. doi: 10.1021/acs.jpca.1c03454 (PMC8279642; doi:10.1021/acs.jpca.1c03454)
Supplement: Supplementary file 1 — jp1c03454_si_001.pdf [file jp1c03454_si_001.pdf]

Complex reaction network thermodynamic and kinetic auto-construction based on *ab initio* statistical mechanics: A case study of O_2 activation on Ag_4 clusters

Weiqi Wang,* Xiangyue Liu, and Jesús Pérez-Ríos

Fritz-Haber-Institut der Max-Planck-Gesellschaft, Faradayweg 4-6, D-14195 Berlin, Germany

E-mail: wang@fhi-berlin.mpg.de

Method details

In the first step, we employ *ab initio* REMD simulation for an efficient sampling of the configurational space, from which a set of trajectories $\{\boldsymbol{x}_{i,j}, i \in [1, N_{atom}], j \in [1, N_{step}]\}$ can be obtained. Here $\boldsymbol{x}_{i,j}$ is the Cartesian coordinate of an atom, N_{atom} is the number of atoms in the system, and N_{step} is the number of MD steps in the trajectory.

Then the sampled structures $\{\boldsymbol{x}_{i,j}\}$ are featurized from the high-dimensional Cartesian coordinates to histogram coordination numbers $\{\mathbf{HCN}_j, j \in [1, N_{step}]\}$ by Eq. (4) as

$$\{\mathbf{HCN}_j\} = \{(h_i, ..., h_{N_{atom}})_j\}. \tag{S1}$$

where h_i is the accumulated number of atoms for a specific coordination environment defined in Eq. (4).

In this work, the HCN representations of the 11 typical isomers described in Fig. 3 are identified and labeled as $\{\boldsymbol{G}_k, k \in [1, 11]\}$. Based on the HCNs $\{\boldsymbol{G}_k\}$ of these typical isomers, the HCN vectors of the sampled configurations $\{\mathbf{HCN}_j\}$ are coarse-grained and transferred to $\{g_j, g \in \{\boldsymbol{G}_k\}\}$ by

$$g_j = \arg\min_k \{||\mathbf{HCN}_j - \boldsymbol{G}_k||, k \in [1, 11]\}. \tag{S2}$$

Then the free energies of the 11 typical isomers can be calculated by substituting $\boldsymbol{x}_{kn}$ in Eq. (8) to g_j. The transition matrix of coarse-grained isomers is calculated by Eq. (9) and the Markov state model is optimized from the transition matrix using the maximum likelihood algorithm,[1]

$$\hat{M} = \arg\max_{\mathbf{TM}} p(\mathbf{CM}|\mathbf{TM}), \tag{S3}$$

where $\mathbf{CM}$ is the count matrix to record the number of transitions between Markov states, and $\mathbf{TM}$ is the transition matrix obtained from Eq. (9). When optimizing the Markov state

model, the detailed balance condition should be satisfied.

The transition probabilities between the coarse-grained states can be obtained from the optimized Markov state model. The normalized activation probabilities are obtained by multiplying the stationary distribution probabilities by the inherent activation probabilities, defined in this work as the ratio of Ag_4O_2 configurations that activate O_2.

To study the reaction pathways of the O_2 activation, the dimension reduction method sketch-map is employed, which transfers the 4-dimensional $\{\mathbf{HCN}_j\}$ to 2-dimensional representation by minimizing Eq. (7).

The cutoff function in sketch-map is used to separate close pairs, medium pairs and long pairs of configurations. As shown in Fig. S1, the cutoff function clearly separate the first peak, which is the fluctuation of the system around the local minimum. After a fast increasing, the cutoff function slowing increases from the second peak to the last. The feature of the cutoff function assists the non-linear projection to sense the similarities of the configurations. The parameters of the cutoff function are: $\sigma = 0.25$, $a = 15.0$, $b = 1.5$.

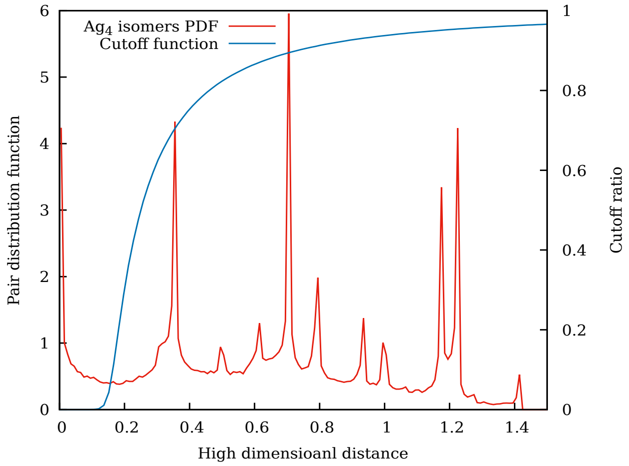

Figure S1: Pair distribution function of Ag_4 clusters and the cutoff function for sketch-map reduction.

With the resultant 2-dimensional vectors $\{S_j\}$, the free energy surface can be calculated by substituting $\boldsymbol{x}_{kn}$ in Eq. (8) to $\{S_j\}$, while Markov state model is estimated based on $\{S_j\}$.

With transition path theory introduced in Sec. 2.4.2, the total reactive flux from a defined reactant $\boldsymbol{\alpha}$ to a defined product $\boldsymbol{\beta}$ can be calculated based on 2-dimensional represented

Markov state model via

$$f_{\mathrm{tot}}^{\boldsymbol{\alpha\beta}} = \sum_{v\in\boldsymbol{\alpha}, w\notin\boldsymbol{\alpha}} f_{v,w}^{\boldsymbol{\alpha\beta}}, \tag{S4}$$

where v and w are the intermediate states in the pathway between $\boldsymbol{\alpha}$ and $\boldsymbol{\beta}$. The reactive flux $f_{v,w}^{\boldsymbol{\alpha\beta}}$ is obtained from the MD trajectories by counting the transitions from $\boldsymbol{\alpha}$ to $\boldsymbol{\beta}$.

The reaction rate from the reactant $\boldsymbol{\alpha}$ to the product $\boldsymbol{\beta}$ can be calculated by

$$k_{\boldsymbol{\alpha\beta}} = \frac{f_{\mathrm{tot}}^{\boldsymbol{\alpha\beta}}}{\sum_{v\in I} \pi_v q_v^-}, \tag{S5}$$

where I is a set of the intermediate states, π_v is the stationary distribution probability of state v, and q_v^- is the committor that indicates the probability reaching state $\boldsymbol{\alpha}$ at state v. The mean first passage time can be calculated from the inverse of the reaction rate.

References

(1) Prinz, J.-H.; Wu, H.; Sarich, M.; Keller, B.; Senne, M.; Held, M.; Chodera, J. D.; Schütte, C.; Noé, F. Markov models of molecular kinetics: Generation and validation. *J. Chem. Phys.* **2011**, *134*, 174105.
